# Source Apportionment and Human Health Risks of Potentially Toxic Elements in the Surface Water of Coal Mining Areas

**DOI:** 10.3390/toxics12090673

**Published:** 2024-09-15

**Authors:** Yuting Yan, Yunhui Zhang, Zhan Xie, Xiangchuan Wu, Chunlin Tu, Qingsong Chen, Lanchu Tao

**Affiliations:** 1 Yibin Research Institute, Southwest Jiaotong University, Yibin 644000, China; 2 Faculty of Geosciences and Engineering, Southwest Jiaotong University, Chengdu 611756, China; 3 Sichuan Province Engineering Technology Research Center of Ecological Mitigation of Geohazards in Tibet Plateau Transportation Corridors, Chengdu 611756, China; 4 Kunming General Survey of Natural Resources Center, China Geological Survey, Kunming 650100, China; 5 Innovation Base for Eco-Geological Evolution, Protection and Restoration of Southwest Mountainous Areas, Geological Society of China, Kunming 650100, China

**Keywords:** potential toxic elements, non-carcinogenic risk, carcinogenic risk, positive matrix factorization

## Abstract

Contamination with potentially toxic elements (PTEs) frequently occurs in surface water in coal mining areas. This study analyzed 34 surface water samples collected from the Yunnan–Guizhou Plateau for their hydrochemical characteristics, spatial distribution, source apportionment, and human health risks. Our statistical analysis showed that the average concentrations of PTEs in the surface water ranked as follows: Fe > Al > Zn > Mn > Ba > B> Ni > Li > Cd > Mo > Cu > Co > Hg > Se > As > Pb > Sb. The spatial analysis revealed that samples with high concentrations of Fe, Al, and Mn were predominantly distributed in the main stream, Xichong River, and Yangchang River. Positive matrix factorization (PMF) identified four sources of PTEs in the surface water. Hg, As, and Se originated from wastewater discharged by coal preparation plants and coal mines. Mo, Li, and B originated from the dissolution of clay minerals in coal seams. Elevated concentrations of Cu, Fe, Al, Mn, Co, and Ni were attributed to the dissolution of kaolinite, illite, chalcopyrite, pyrite, and minerals associated with Co and Ni in coal seams. Cd, Zn, and Pb were derived from coal melting and traffic release. The deterministic health risks assessment showed that 94.12% of the surface water samples presented non-carcinogenic risks below the health limit of 1. Meanwhile, 73.56% of the surface water samples with elevated As posed level III carcinogenic risk to the local populations. Special attention to drinking water safety for children is warranted due to their lower metabolic capacity for detoxifying PTEs. This study provides insight for PTE management in sustainable water environments.

## 1. Introduction

Clean fresh water is an indispensable resource for life on Earth [1,2]. Surface water, one of the Earth’s vital freshwater resources, plays a vital role in the global drinking water supply [3,4,5]. However, this resource, including lakes, rivers, and other water bodies, is more easily contaminated than groundwater because it is directly exposed to the external environment. Pollutants such as agricultural fertilizers [6], pesticides [7], industrial discharge [8], and urban pollution [9] can deteriorate surface water quality. Among these contaminants, potentially toxic elements (PTEs) from serial industrial and urbanization activities may infiltrate and accumulate in surface water [10]. PTEs comprise metals, semi-metals, and non-metals such as Al, V, Cr, Mn, Fe, Co, Ni, Cu, Zn, As, Se, Mo, Cd, Sb, Ba, Li, B, Hg, and Pb due to their potential toxicity [11,12,13]. PTE pollution has become a global environmental issue [14,15]. Numerous cases of PTE contamination have been widely reported around the world, including in China [10,16], the United States [17], and Europe [12,18]. For instance, 41% and 8% of water samples from Kumaun Himalaya, India, had Cr and Pb concentrations above World Health Organization (WHO)’s drinking limits [19]. Moreover, the Fe, Mn, Pb, Cr, and Tl concentrations in water around a pyrite mine in China also exceeded the WHO levels [20]. Therefore, identifying the sources of PTEs and assessing their potential human health risks are essential to protecting surface water from PTE pollution.

Some toxic metallic, non-metallic, and semi-metallic elements in water can pose hazards to the human body at elevated concentrations [11]. The term “heavy metals” has been used to refer to these toxic elements for decades. The term “heavy metals” is controversial, so the term PTEs has been suggested to refer to these elements instead [21]. Elevated concentrations of PTEs are often attributed to geogenic conditions such as strata enrichment or mineral dissolution. In addition, anthropogenic activities like mining, coal combustion, industrial discharge and so on play a vital role in transporting PTEs to surface water [22,23,24]. To date, extensive research has focused on identifying and quantifying these various sources of PTE pollution. Consequently, source apportionment methods like principal component analysis (PCA), absolute principal component scores–multiple linear regression (APCS-MLR), UNMIX, and positive matrix factorization (PMF) were developed for source analysis [14,25,26]. Among these, the PMF model stands out for its ability to explain physical meaning, handle diverse datasets, resist noise, and operate without the need to predefine the number of pollution sources [25,27]. Owing to its capacity to determine sources and quantify their contributions accurately, the PMF model was selected as the source apportionment model in this study.

Exposure to high levels of PTEs can lead to serious health issues, such as gastrointestinal disorders, nervous system disease, and even cancer [28,29,30]. These elevated concentrations of PTEs are more pronounced in mining areas, posing significant hazards to human health [24,31]. Therefore, understanding PTE risks and developing effective pollution prevention measures are crucial for safeguarding the health of residents near mining sites. To evaluate these hazardous effects, the United States Environmental Protection Agency (USEPA) proposed a health risk assessment model, which has become a fundamental tool for health risk appraisal [32]. This model accounts for variables containing exposure time, exposure pathways, PTE concentrations, human age, and body weight [33]. It classifies risks into non-carcinogenic and carcinogenic categories based on the carcinogenicity of PTEs [34]. Non-carcinogens refer to compounds that do not have harmful effects for humans below a certain exposure dose, such as Fe, Mn, Cu, Zn, Al, As, Se, Cd, Li, B, Ba, Sb, Ni, Co, and Mo [32]. Carcinogens are compounds that induce carcinogenic reactions at all doses greater than zero, such as As and Cr^6+^ [35]. Furthermore, sensitivity analysis has become increasingly popular in recent research to understand the impacts of risk variables on health risk assessment models [5].

The study area is situated in the Wudu River Basin on the Yunnan–Guizhou Plateau in southwest China. The Xichong River, Yangchang River, and Xichang River serve as essential water resources for drinking, industrial, and irrigation purposes [36]. However, this study area is also a significant coal mine production and supply base. As a result, the PTEs in surface water may reach elevated concentrations, posing health risks to residents. Hence, tracing the sources of PTEs and assessing their health risks is crucial for the health and well-being of the local population. Nonetheless, many studies on surface water source apportionment have overlooked the influence of geological and anthropogenic factors related to groundwater and mine water. Moreover, existing studies in this study area have primarily focused on hydrochemical analysis [36], with limited attention to the source apportionment of PTEs. To bridge this gap, this study identifies the sources of PTEs by integrating analyses of groundwater and mine water samples. Therefore, this study aims to (1) analyze the hydrochemical characteristics and spatial distribution through box plots and spatial distribution maps, (2) identify the sources of potentially toxic metals using the positive matrix factorization (PMF) model, and (3) assess both non-carcinogenic and carcinogenic human health risks utilizing the deterministic health risk model.

The research findings offer new insights into preventing surface water contamination and developing sustainable surface water management strategies.

## 2. Study Area

**Figure 1:**
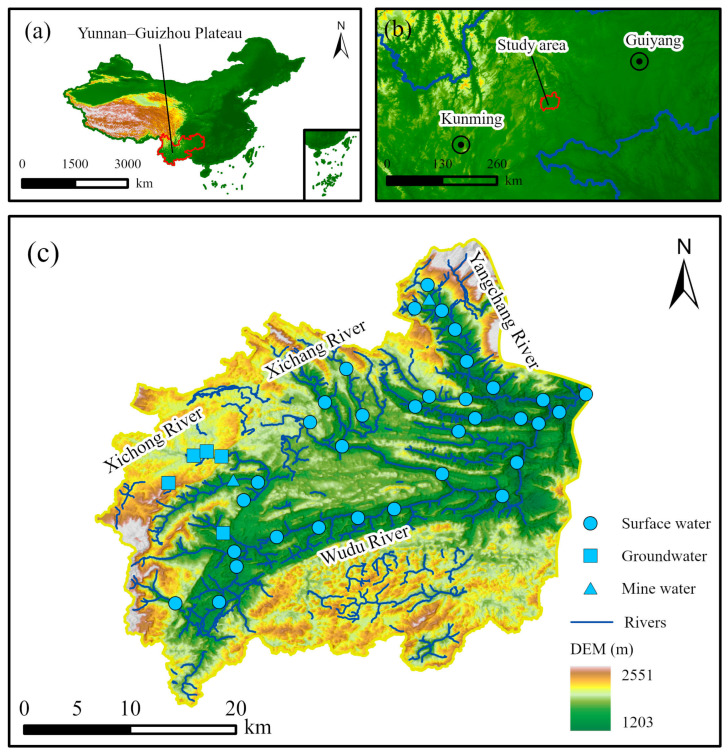
(**a**) Location of the Yunnan–Guizhou Plateau in China, (**b**) location of the study area in the Yunnan–Guizhou Plateau, and (**c**) location of surface water, groundwater, and mine water sampling sites in the study area (sample size = 34).

The study area is situated on the Yunnan–Guizhou Plateau in southern China, encompassing latitudes 104°30′45″ N–104°55′56″ N and longitudes 25°36′5″ E–25°59′47″ E (Figure 1). The terrain is a plateau mountain, characterized by higher elevations in the northern and southern regions, with lower elevations in the central part (Figure 1c). The Wudu River, the primary river system in the study area, includes three tributaries: the Xichong River, the Yangchang River, and the Xichang River. The main stream extends for 58.71 km, and the catchment area covers approximately 1109.09 km^2^. The study area is characterized by a subtropical monsoon humid climate, with the majority of rainfall occurring between June and September, and an average annual precipitation of 615.5 mm. The average monthly temperature reaches its lowest value in January, at 17.1 °C, and peaks in August, at 24.3 °C.

The northern part of the study area is dominated by carbonate rocks from the Triassic Middle Guanling Formation, Falang Formation, and Lower Jialingjiang Formation (Figure S1). The northeast and southwest parts are mainly composed of clastic rocks from the Lower Triassic Feixianguan Formation, the Upper Permian Emeishan Basalt, and the Longtan Formation. Notably, the Longtan Formation of the Upper Permian contains abundant coal resources, with an average thickness of 341 m. The southern part of the study area features carbonate rocks of the Carboniferous and Devonian strata. Karst landforms are developed in the study area. The groundwater is primarily carbonated karst water, with the water table depth being less than 50 m. Spring and underground river flows range from 10 to 2000 L/s. The groundwater is mainly discharged along rivers and springs. The average annual flow of the Wudu River’s main stream is 39,500 L/s.

A built area was distributed across the study area, comprising 14.72% of the total area (Figure S2). The built area included a major road, rail networks, and large homogenous impervious surfaces [37], corresponding to artificial surfaces in Corine Land Cover, as shown in Table S3. The crops were distributed in the southern part of study area, covering 7.9% of the total area. The Wudu River served as the primary source for industrial and irrigation activities, while groundwater was the main source of drinking water for residents. There are about total 76 coal mines in the study area, which are distributed along the Xichong River, the Yangchang River, and the main stream of the Wudu River (Figure S3).

## 3. Materials and Method

### 3.1. Field Sampling and Laboratory Analysis

A total of 34 surface water samples were collected from the Wudu River in July 2022 (Figure 1c). To trace the source of surface water, additional samples were obtained, including 5 groundwater samples and 2 mine water samples from springs, underground rivers, and coal mines. Mine water samples from the Xichong River and the Yangchang River were collected from abandoned coal mines and mine water treated during coal mine production, respectively. The surface water was collected directly from the river, and groundwater and mine water were extracted by a water pump. Before sampling, wells were flushed until the electrical conductivity stabilized within ±10 of three consecutive measurements. Each sampling bottle was rinsed three times with the sampling water prior to collection. After filtration through a 0.45 μm microporous filter membrane, 2.5 L of water was collected at each site and stored in polyethylene bottles. A total of 5 mL of concentrated nitrite acid was added into the water samples for analyzing Fe, Mn, Cu, Zn, Al, Pb, and Cd, while 5 mL of concentrated hydrochloric acid was added into the water samples for analyzing Hg, As, Se, and Sb. We added a drop of sodium hydroxide solution with a concentration of 10 g/L to the water sample for analyzing Cr. Additionally, one bottle of pure water was collected to verify any potential contamination during pre-treatment and analysis. After sampling, all samples were stored in the fridge to be kept at 4 °C. Then, all samples were transported to the Kunming Comprehensive Natural Resources Survey Center, China Geological Survey (CGS), for testing. Trace elements, including Fe, Mn, Cu, Zn, Al, Hg, As, Se, Cd, Pb, Li, B, Ba, Sb, Ni, Co, Mo, Cr, Ag, and Be were analyzed using an atomic absorption spectrometer (). The laboratory determination was based on the analysis protocol “Method for analysis of groundwater quality” (DZ/T 0064-2021) and “Environmental quality standards for surface water” (GB 3838-2002) [38,39]. The Cr, Ag, and Be concentrations were below the detection limits. Certified reference materials (CRM) including GNM-M28593-2013, GNM-M28593-2013, and GBW (E) 083186a-2 and collected water samples were analyzed simultaneously for quality assurance (QA). A test result was considered acceptable if each component fell within twice the uncertainty of the CRM standard’s recommended value. The relative standard deviation (RSD) for random sample repeat analysis was controlled within ±5%. Full-process blank tests were conducted to ensure quality control (QC). When the sample value was less than 20*MDL, the blank value needed to be less than 2*MDL. When the sample determination value was greater than 20*MDL, the blank determination value needed to be less than 10*MDL.

### 3.2. Positive Matrix Factorization (PMF) Model

The positive matrix factorization (PMF) model, originally developed by Paatero and Tapper [40] is an innovative inceptor model. The PMF model factorizes concentration matrix *X* sing the least square methods. When the *Q* value reaches its minimum, the concentration matrix *X* is decomposed into two factor matrixes—factor contribution matrix *G* and factor profile matrix *F*—along with a residual matrix *E*, as shown in Equations (1) and (2).

(1) $$X_{\left(n\times m\right)}=G_{\left(n\times p\right)}*F_{\left(p\times m\right)}+E_{\left(n\times m\right)}$$

(2) $$Q=\sum_{i=1}^{m}\sum_{j=1}^{n}(\frac{x_{ij}-\sum_{k=1}^{p}g_{ik}f_{kj}}{u_{ij}}{)}^{2}$$

where *n* represents the number of samples, *m* refers to the number of hydrochemical parameters, *p* indicates the number of resolved sources, and *u_ij_* denotes the uncertainty of the j hydrochemical parameters in the *i* sample.

The uncertainty of the concentration of each hydrochemical parameter was calculated by Equation (3).

(3) $$u_{ij}=\left\{\begin{array}{c} \frac{5}{6}\times MDLj,\;x_{ij}\leq {MDL}_{j}\\ \sqrt{{\left({EF}_{j}\times x_{ij}\right)}^{2}+({\left(0.5\times {MDL}_{j}\right)}^{2}},\;x_{ij}>{MDL}_{j} \end{array}\right.$$

where *MDLj* represents the method detection limit for parameter *j* in Table S1, and *EFj* refers to the error fraction of parameter *j*. The error fraction (*EF*) was estimated based on the standard deviation and missing proportions of hydrochemical data.

### 3.3. Deterministic Human Health Risk Assessment

The human health risk assessment model includes deterministic and probabilistic approaches. Due to the limited hydrochemical dataset in this study, which consisted only of 34 samples, it was not feasible to determine the distribution type of the hydrochemical parameters. Consequently, only the deterministic human health risk model was employed to quantify the human health risks associated with surface water. Chronic daily ingestion (*CDI*) through oral ingestion was calculated based on concentrations of PTEs (*C_w_*) and various exposure parameters (*IR*, *EF*, *BW*, and *AT*) using Equation (4). The hazard quotient (*HQ*), which represents the non-carcinogenic risk, was derived from the ratio of *CDI* and the reference dose (*RfD*) (Equation (5)). The cancer risk (*CR*) due to carcinogenic PTEs was obtained by multiplying the CDI by the slope factor (*SF*) (Equation (6)). The hazard index (*HI*) and cumulative cancer risks (*CCR*) were calculated as the sum of *HQ* and *CR* for multiple PTEs, respectively (Equations (7) and (8)). The populations in the assessment were divided into three groups—children, women, and men—for the health risk assessment. The definitions and values of the calculating parameters used for different populations are provided in Table S4.

(4) $$CDI=\frac{C_{W}\times IR\times EF\times ED}{BW\times AT}$$

(5) $$HQ=\frac{CDI}{RfD}$$

(6) CR=CDI×SF

(7) $$HI=\sum_{j=1}^{m}{HQ}_{j}$$

(8) $$CCR=\overset{m}{\underset{j=1}{\sum }}CR$$

where the *AT* of non-carcinogenic effects is 365 × *EF*, and the *AT* of carcinogenic effects is life expectancy × *EF*.

Non-carcinogenic risks can be divided into two levels based on threshold 1: level I (≤1) and level II (>1). Level I indicates that the exposure quality does not exceed the adverse reaction threshold. On the contrary, level II indicates that the exposure quality exceeds the threshold and should be of concern. Carcinogenic risks can be divided into five levels: level I (<10^−6^), level II (1 × 10^−6^–1 × 10^−5^), level III (1 × 10^−5^–1 × 10^−4^), level IV (1 × 10^−4^–1 × 10^−3^), and level V (>1 × 10^−3^) (Table S5). Level I indicates that the cancer risks are low; level II and level III indicate that there are certain carcinogenic risks that should be paid attention to; and level IV and level V indicate that the risk of cancer is higher, and the water is not suitable for drinking.

PTEs are potentially toxic and can harm the human body, causing various diseases [41]. The Integrated Risk Information System (IRIS) has investigated the non-carcinogenic effects of PTEs in the environment [42]. Mn and Se can damage the nervous system, while Cd, Ba, and Mo can damage the urinary system. Zn and Sb can cause blood problems, while As can cause cardiovascular and skin problems. B and Ni can decrease body weight, particularly in infants. Fe, Cu, Al, Li, and Co have been identified as pathogenic metals in recent scientific research [43,44,45]. Therefore, the deterministic non-carcinogenic risk of these PTEs was assessed in this study. The reference doses (*RfDs*) of non-carcinogenic PTEs are shown in Table 1.

### 3.4. Statistical Analysis

The NumPy and Panda libraries in Python were employed for the statistical analysis of descriptive statistics and deterministic human health risks. The Matplotlib and seaborn libraries in Python 3.10 were used to draw statistical graphs. ArcGIS Pro 3.0 was utilized to generate spatial distribution maps of concentrations and health risks. The PMF 5.0 software was used to apply the PMF model.

## 4. Results and Discussion

### 4.1. Hydrochemical Characteristics of Surface Water

**Figure 2:**
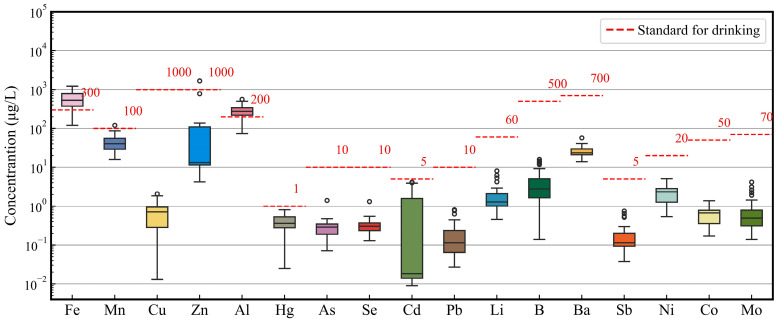
Box plots with the standard of potentially toxic elements for drinking surface water (sample size = 34).

Descriptive statistics and box plots with drinking water standards for PTEs in surface water are summarized in Table S6 and Figure 2. The average concentrations of PTEs are ranked as follows: Fe > Al > Zn > Mn > Ba > B> Ni > Li > Cd > Mo > Cu > Co > Hg > Se > As > Pb > Sb. According to the class III drinking water standards established by China [46], 79.41% of the samples (27 samples) exceeded the permissible limits for Fe and Al. Only 2.94% of the samples (one sample) showed concentrations of Mn and Zn that exceeded the standard. The maximum concentrations of Hg and Cd were 0.81 μg/L and 4.13 μg/L, approaching the standards of 1 and 5 μg/L. The coefficient of variation (CV) of the PTEs was ranked as follows: Zn > Cd > Mo > Pb > B > Sb > Li >Cu > As > Se > Fe > Co > Mn > Hg > Ni > Al > Ba. The high CV (274.42%) of the Zn concentrations indicated that there were significant differences in the Zn concentrations between the three tributaries and the main stream of the Udu River.

### 4.2. Spatial Distribution Maps of Surface Water

**Figure 3:**
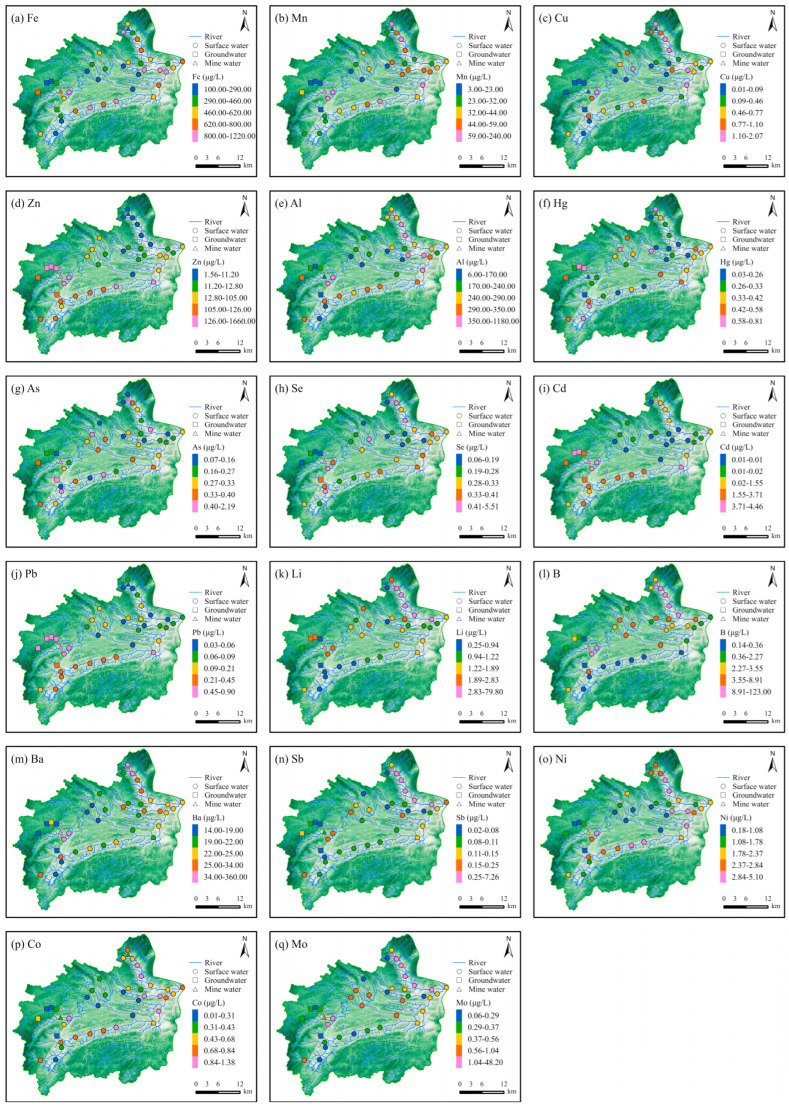
Spatial distribution map of concentrations of potentially toxic elements: (**a**) Fe, (**b**) Mn, (**c**) Cu, (**d**) Zn, (**e**) Al, (**f**) Hg, (**g**) As, (**h**) Se, (**i**) Cd, (**j**) Pb, (**k**) Li, (**l**) B, (**m**) Ba, (**n**) Sb, (**o**) Ni, (**p**) Co, and (**q**) Mo (sample size = 34).

To show the difference in the PTE concentrations, the “GraduatedColorsRenderer” and “Quantile” principle in ArcGIS Pro were used to classify the surface water samples. As a result, each range had different widths but the same observation frequency. The spatial distribution of the Fe, Mn, Cu, Al, Ba, Ni, and Co concentrations followed a similar trend, with elevated levels primarily distributed in the main stream, Xichong River, and Yangchang River (Figure 3a–c,e,m,o,p). The spatial distribution of Zn, Cd, and Pb exhibited similar patterns, with higher concentrations in the main stream and Xichong River (Figure 3d,i,j). Notably, the Cd concentrations in the surface water of the Yangchang River were relatively higher than those in the Xichong River. The spatial distribution of Se, Li, B, Sb, and Mo exhibited similar characteristics, with the concentrations in the tributaries being significantly higher than those in the main stream (Figure 3h,k–m,q). Additionally, the concentrations of Se and Mo in the mine water were similar to those in the surface water but higher than in the groundwater.

The concentrations of Cu and Co in two mine water samples were low, whereas the concentrations of As, Se, Cd, Li, B, Ba, Ni, and Mo were elevated. Additionally, the concentrations of Fe, Mn, Zn, and Pb in the mine water from an abandoned coal mine were higher than those in a productive coal mine. In contrast, the concentrations of Al, Hg, and Sb were higher in the active coal mine but lower in the abandoned one.

### 4.3. Source Apportionment for PTEs

**Figure 4:**
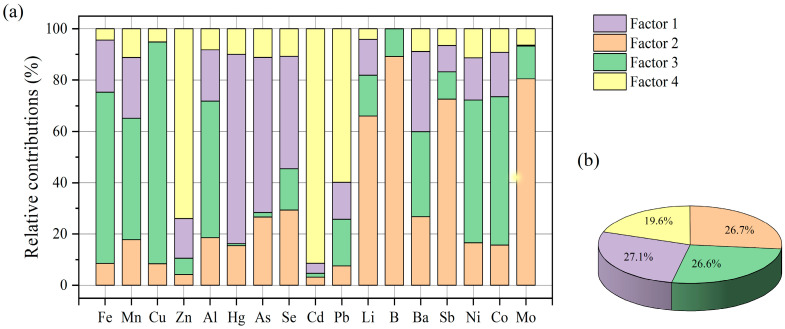
Source contributions of PTEs based on the PMF model: (**a**) relative contributions of PTEs to PMF factors and (**b**) average contributions of PMF factors (sample size = 34).

The PMF model was applied to analyze the PTEs in surface water, and the debugging details for ensuring a reliable source recognition effect are presented in Text S1, Tables S1 and S2. Four factors were identified, contributing 27.1%, 26.7%, 26.6%, and 19.6% to the total source attribution, respectively (Figure 4).

Factor 1 was represented by Hg, As, and Se. The concentrations of Hg, As, and Se in surface water were higher than in groundwater, indicating that the concentrations of Hg, As, and Se were not controlled by geological factors (Figure 3f–h). Hg, As, and Se are common pollutants in mining industry wastewater [47]. The maximum concentration of Hg was 0.81 μg/L, close to the drinking water limit of 1 μg/L (Table S6 and Figure 2). However, the Hg concentrations in mine water were 0.30 and 0.17 μg/L, lower than those in surface water. Therefore, the excessive Hg originated from the wastewater of coal preparation plants, not from the coal mine water. The max concentrations of As and Se were 1.41 and 1.32 μg/L, respectively, which were lower than the drinking water limit of 10 μg/L. In addition, the concentrations of As and Se in mine water were higher than those in surface water. Consequently, the low concentrations of As and Se in surface water came from coal mine wastewater meeting discharge standards. Factor 1 reflects the wastewater discharged from coal preparation plants and coal mine water.

Factor 2 was represented by Mo, Li, and B. The concentrations of Mo, Li, and B in surface water were lower than their drinking water limit (Figure 2). The surface water samples with elevated concentrations of Mo, Li, and B were distributed in the coal-bearing strata (Figure 3l,q,k). Mo, Li, and B are likely to be present in the clay minerals of coal [48,49,50]. Therefore, the low concentrations of Mo, Li, and B in surface water were due to the dissolution of clay minerals. Factor 2 represents the dissolution of clay minerals in coal seams.

Factor 3 was presented by Cu, Fe, Al, Mn, Co, and Ni. The samples with elevated concentrations of Cu, Fe, Al, Mn, Co, and Ni were distributed within the Upper Permian Longtan Formation (Figure 3a–c,e,o,p and Figure S1). Geologically, the Longtan Formation is a distinctive formation, consisting of sedimentary rocks and coal seams. Kaolinite (Al_4_[Si_4_O_10_] (OH)_8_) and illite are abundant in sedimentary rocks, whereas coal seams are rich in chalcopyrite (CuFeS_2_) and pyrite (FeS_2_). Moreover, multi-stage hydrothermal processes can lead to the enrichment of rare metals (such as Co and Ni) in coal seams during the diagenetic process [51]. Therefore, Factor 3 reflects the dissolution of chalcopyrite, pyrite, kaolinite, illite, and mineral-enriched Co and Ni formed in multi-stage hydrothermal processes during sedimentation.

Factor 4 is predominantly influenced by Cd, Zn, and Pb. Elevated concentrations of Cd, Pb, and Zn are distributed along the Xichong and Wudu Rivers (Figure 3d,i,j). Numerous coal melting and processing facilities are located in the Xichong River catchment. Cd is usually present in trace forms in coal [52]. Additionally, Cd is often released into wastewater during high-temperature smelting processes [53]. Therefore, the excessive accumulation of Cd is the result of extensive coal combustion. Emissions from transportation can lead to elevated concentrations of Zn and Pb [54]. Frequent mining activities have resulted in a significant transportation demand in the Xichong and Wudu River catchments. The combustion of gasoline and natural gas has led to the accumulation of Cd, Zn, and Pb in natural water bodies. Therefore, Factor 4 was identified as originating from industrial and traffic sources.

Nisar identified controlling factors of PTEs in the groundwater of northern Pakistan using the PMF model [55]. Four factors were included: mining/agricultural discharge (As and Fe), industry (Zn), municipal solid waste (Cd and Cu), and domestic sewage (Pb and Fe). Nisar’s study simply attributed both As and Fe concentrations to coal mining activities. In this study, compared to the As concentration in mine water, the As in surface water is supposed to come from treated mine water. Because the Fe concentration far exceeds the limit value, Fe concentrations tend to come from the pyrite exposed in coal seams. In this study, more specific sources of As and Fe are proposed, based on mine water, groundwater, and the drinking limit.

### 4.4. Deterministic Human Health Risk Assessment of PTEs

#### 4.4.1. Non-Carcinogenic Human Health Risks

**Figure 5:**
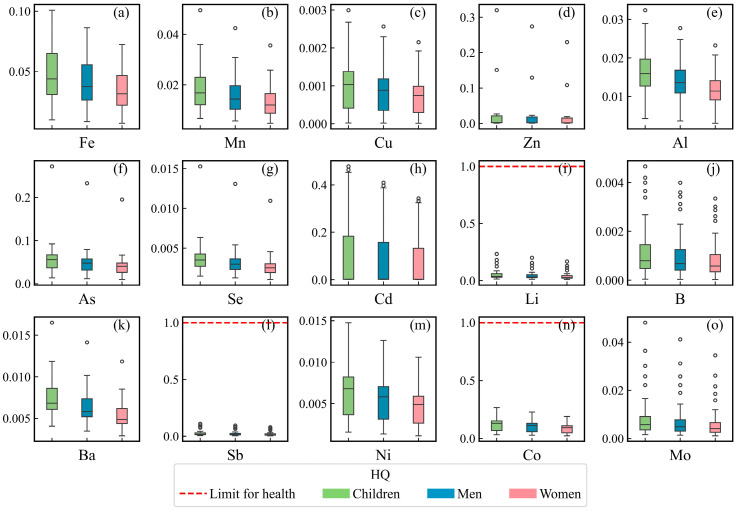
Non-carcinogenic health risks of surface water to children, men, and women: (**a**) Fe, (**b**) Mn, (**c**) Cu, (**d**) Zn, (**e**) Al, (**f**) As, (**g**) Se, (**h**) Cd, (**i**) Li, (**j**) B, (**k**) Ba, (**l**) Sb, (**m**) Ni, (**n**) Co, and (**o**) Mo (sample size = 34).

**Figure 6:**
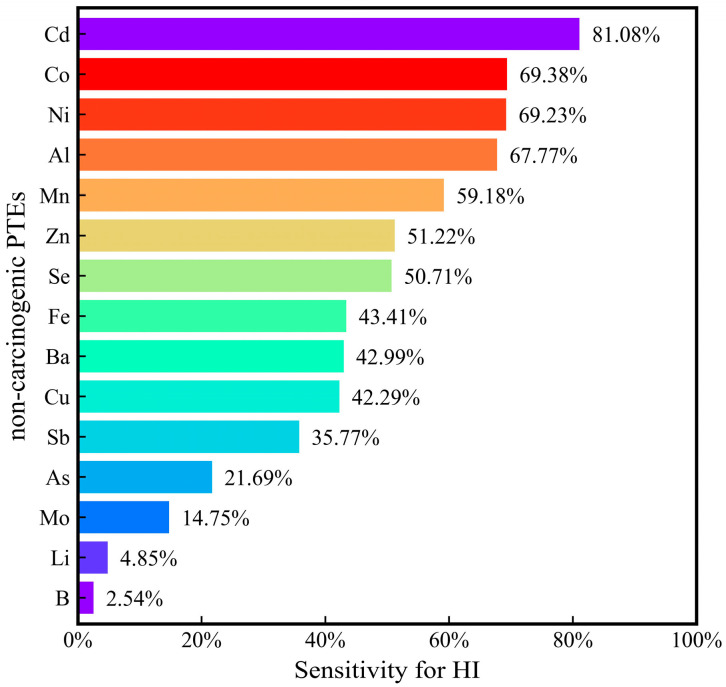
Sensitive non-carcinogenic PTE ranking for HI.

The hazard quotient (HQ) of individual PTEs to children, men, and women was less than 1, indicating that the non-carcinogenic health risks of individual PTEs were within acceptable limits (Figure 5). Sensitivity analyses were performed to identify the sensitivity of the hazard index (HI) to individual PTEs. The HI was highly sensitive to the concentrations of Cd, Co, and Ni, indicating that these concentrations need to be monitored in surface water (Figure 6). The HI was not sensitive to the concentrations of Li and B, indicating that the hazard posed by Li and B was minimal. The non-carcinogenic risks to children were greater than those for men and women, demonstrating that children are more susceptible to the hazards of PTEs in drinking water. This is because children have a lower body weight and a weaker resistance.

**Figure 7:**
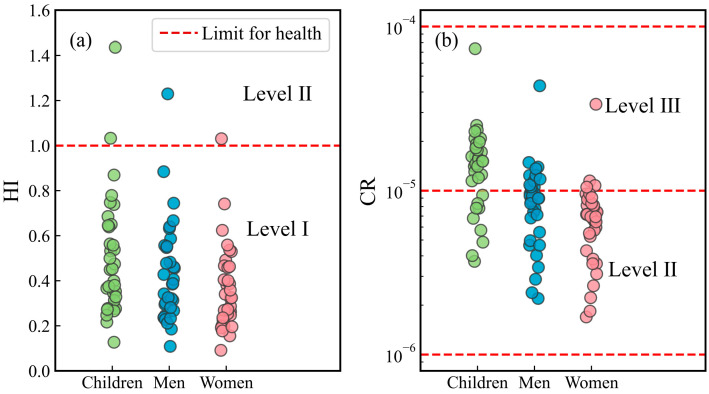
Non-carcinogenic and carcinogenic health risks of PTEs in surface water to children, men, and women: (**a**) hazard index (HI) and (**b**) cancer risk (CR) (sample size = 34).

**Figure 8:**
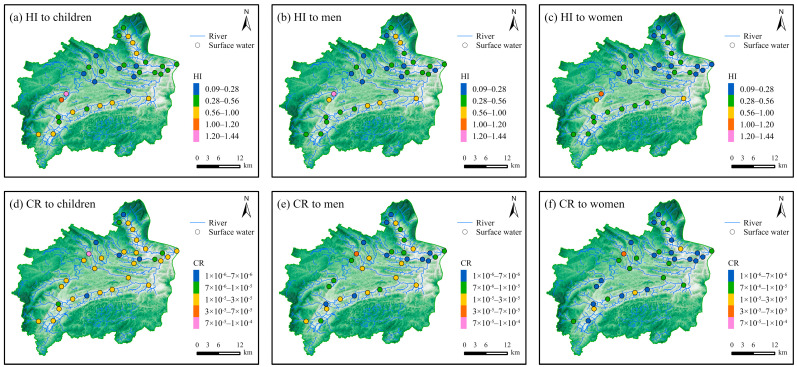
Spatial distribution map of hazard index (HI) and cancer risk (CR) of PTEs in surface water. HI to (**a**) children, (**b**) men, and (**c**) women and CR to (**d**) children, (**e**) men, and (**f**) women (sample size = 34).

The HI was greater than 1 at two sample sites, both located along the Xichong River (Figure 7a and Figure 8a–c). There were several coal mines and coking plants distributed in these sites (Figure S3). Meanwhile, this area was the population center (Figure S2). Coal mining and frequent human activities have increased PTE concentrations in the surface water in this area. It is necessary to decrease the PTE concentrations in the Xichong River. Additionally, surface water samples with a relatively high HI were distributed in the main streams of the Udu and Yangchang Rivers. Elevated PTE concentrations of surface water in these areas need continuous monitoring.

#### 4.4.2. Carcinogenic Human Health Risks

The As is a carcinogenic element and can cause skin cancer in humans [34]. The deterministic carcinogenic risks of the As in the surface water to children, men, and women were evaluated in this study. The CR of As for all populations was below the limit value of 1 × 10^−4^, indicating that the cancer risk from surface water was low (Figure 7b). A total of 73.5% of the samples, distributed across surface water systems, posed carcinogenic risks of level III to the various populations (Figure 8d–f, Table S4). Notably, one sample from the Xichong River had the highest CR, with the CR for children approaching the limit value of 1 × 10^−4^. Hence, special attention should be given to these samples, particularly the one with the highest CR. Furthermore, 26.5% of the samples had a carcinogenic risk of level II, indicating that these samples were suitable for drinking water. Similar to the non-carcinogenic risks, the carcinogenic risks to children were higher than those to men and women. Thus, the safety of children’s drinking water needs to be emphasized. The concentration of As in surface water directly determined the carcinogenic risk level in the study area. Therefore, controlling the As concentrations in surface water is necessary for preventing carcinogenesis in local residents.

### 4.5. Suggestions for Ensuring Human Health

The surface water in the study area was enriched with excessive levels of Fe, Mn, and Al. Additionally, relative high concentrations of Cd and As in the surface water posed significant non-carcinogenic and carcinogenic risks to local residents, respectively. To ensure water environmental security and protect human health, the following measures are recommended.

(1) Water quality and potability: Elevated concentrations of Fe, Mn, and Al concentrations diminished the suitably for drinking. Fe, Mn, and Al mainly dissolved from minerals associated with coal seams. Therefore, surface water must be treated through a reverse-osmosis system or a home water purifier prior to consumption.

(2) Non-carcinogenic risks of Cd: Excessive Cd originated from coking plants produced most of the HI. The surface water samples with excessive non-carcinogenic risks were distributed near the Xichong River. Therefore, coking plants should implement advanced purification systems to effectively treat exhaust gas and wastewater.

(3) Carcinogenic risks of As: Excessive As concentrations threaten the water ecosystem and human body due to its high carcinogenicity. As contamination in this study was primarily linked to mine water discharged from coal mines, it is essential for coal mining companies to adopt effective water treatment techniques to remove As from mine water.

## 5. Conclusions

In this study, a total of 34 surface water samples, 5 groundwater samples, and 2 mine water samples were collected from the Yunnan–Guizhou Plateau in southern China. The analysis focused on the hydrochemical characteristics, spatial distribution, source contributions, and human health risks associated with PTEs such as Fe, Mn, Cu, Zn, Al, Hg, As, Se, Cd, Pb, Li, B, Ba, Sb, Ni, and Co in surface water. The major conclusions were as follows:

(1) The average concentrations of PTEs in surface water were ranked as follows: Fe > Al > Zn > Mn > Ba > B > Ni > Li > Cd > Mo > Cu > Co > Hg > Se > As > Pb > Sb. Notably, 79.41% of the surface water samples exceeded the drinking water standards for Fe and Zn. Samples with elevated concentrations of Fe, Al, and Mn were predominantly distributed in the main stream, Xichong River, and Yangchang River. Elevated Zn concentrations were primarily detected in the main stream and Xichong River.

(2) The sources of PTEs in surface water were attributed to five factors: Hg, As, and Se in Factor 1 were linked to the discharge of wastewater from coal preparation plants and coal mines; Mo, Li, and B in Factor 2 were associated with the dissolution of clay minerals in coal; Cu, Fe, Al, Mn, Co, and Ni in Factor 3 pertained to the dissolution of chalcopyrite, pyrite, kaolinite, and illite in coal strata; and Cd, Zn, and Pb in Factor 4 were related to elevated concentrations of Cd, Zn, and Pb from coal smelting and traffic emissions.

(3) A total of 94.12% of the surface water samples exhibited total non-carcinogenic risks below the health threshold of 1. Two samples with elevated HI values had been taken from the Xichong River. Moreover, 73.56% of the surface water samples posed a carcinogenic risk of level III to the various populations, necessitating increased scrutiny. Notably, children were found to be more sensitive to elevated PTE concentrations in contaminated surface water than adults.

## Figures and Tables

**Table 1:** The reference dose (RfD) and slope factor (SF) of PTEs for non-carcinogenic and carcinogenic health risk assessment (sample size = 34).

Parameters	Unit	Fe	Mn	Cu	Zn	Al	As	Se	Cd	Li	B	Ba	Sb	Ni	Co	Mo
RfD	mg/(kg·d)	0.7	0.14	0.04	0.3	1	0.0003	0.005	0.0005	0.002	0.2	0.2	0.0004	0.02	0.0003	0.005
SF	kg·d/mg	-	-	-	-	-	1.5	-	-	-	-	-	-	-	-	-

## Data Availability

The data presented in this study are available upon request from the corresponding author.

## Supplementary Materials

The following supporting information can be downloaded at https://www.mdpi.com/article/10.3390/toxics12090673/s1: Figure S1: The geological map of the study area; Figure S2: The land use map of the study area; Figure S3: The location of coal mines in the study area; Table S1: The determined parameters for PMF model running; Table S2: Evaluation results of PMF model based on displacement and bootstrap diagnostics; Table S3: Classification and description of artificial surfaces in Corine Land Cover; Table S4: The definition and deterministic value of calculation parameters in the health risk assessment [32,56,57]; Table S5: The classification criteria of carcinogenic risk for health purposes [34]; and Table S6: Descriptive statistics and drinking standard of potentially toxic elements in surface water (sample size = 34).
