# Supplementary material for: Source Apportionment and Human Health Risks of Potentially Toxic Elements in the Surface Water of Coal Mining Areas
Source: Toxics. 2024 Sep 15;12(9):673. doi: 10.3390/toxics12090673 (PMC11435608; doi:10.3390/toxics12090673)
Supplement: Supplementary file 1 [file toxics-12-00673-s001.zip › toxics-3197114-supplementary.pdf]

Supplementary Materials

Text S1 PMF model running

(1) Parameters debugging: The S/N of each parameter was As (0.7) < Se (0.9) = Sb (0.9) < Pb (1.0) < Cu (1.6) < Cd (1.8) = Ba (1.8) < Li (1.9) < B (2.2) < Fe (2.3) = Mn = Zn = Ni < Mo (2.5) < Co (2.9) < Al (3.0) < Hg (3.6). So, the species of As, Se, Pb and Sb were set as weak categories while others were set as strong categories. By setting the number of factor to 4, Q_{Robust}/Q_{True} reached the minimum convergence value with 20 base runs and the scaled residuals of all species were within the range of -3 to +3. Values of R-squared ranged from 0.10 to 0.99 and were in the order of Se (0.10) < As (0.14) < Zn (0.47) < Ba (0.62) < Cu (0.65) < Mn (0.72) < Fe (0.82) < Al (0.84) < B (0.88) < Hg (0.88) < Sb (0.90) < Co (0.92) < Pb (0.93) < Li (0.93) < Ni (0.97) < Mo (0.98) < Cd (0.99).

(3) Displacement (DISP) and bootstrap (BS) diagnostics: Displacement (DISP) and bootstrap (BS) diagnostics were conducted to assess the stability and efficacy of the PMF model. The results are tabulated in Table S2. The BS diagnostics indicated that the error code was zero, and the largest decrease in Q (-0.015) was less than 1% of Q_{Robust} (2.45). Additionally, no unmapped bootstrap factors were observed in the BS diagnostics, reflecting the reliability of the PMF model.

Table S1 The determined parameters for PMF model running.

Parameter	unit	Fe	Mn	Cu	Zn	Al	Hg	As	Se	Cd	Pb	Li	B	Ba	Sb	Ni	Co	Mo
MDL	μg/L	30	3	0.013	0.2	6	0.01	0.07	0.06	0.006	0.02	0.2	0.14	10	0.018	0.02	0.014	0.063
EF	/	0.3	0.3	0.2	0.3	0.25	0.1	0.1	0.1	0.15	0.2	0.25	0.25	0.35	0.1	0.3	0.2	0.1

Table S2 Evaluation results of PMF model based on displacement and bootstrap diagnostics.

Displacement diagnostics				
	Error code	Largest decrease in Q	Q_{Robust}	1% Q_{Robust}
	0	-0.015	245.1	2.45
Swaps by Factor	0	0	0	0

Bootstrap (BS) diagnostics					
	Factor 1	Factor 2	Factor 3	Factor 4	Unmapped
Boot Factor 1	20	0	0	0	0
Boot Factor 2	0	20	0	0	0
Boot Factor 3	0	0	20	0	0
Boot Factor 4	0	0	3	17	0

Table S3. Classification and description of artificial surfaces in Corine Land Cover.

	Value	Description
Artificial surfaces	1	Continuous urban fabric
	2	Discontinuous urban fabric
	3	Industrial or commercial units
	4	Road and rail networks and associated land
	5	Port areas
	6	Airports
	7	Airports
	8	Dump sites
	9	Construction sites
	10	Green urban areas
	11	Sport and leisure facilities

Table S4 The definition and deterministic value of calculation parameters in the health risk assessment [32,56,57].

Parameter	Definitions	Units	Children	Men	Women
C_w	Chemical concentration in water	mg/L	-	-	-
IR	Inhalation rate	L/hour	1.5	3.62	2.66
EF	Exposure frequency	Days/year	365		
ED	Exposure duration	years	6	30	30
BW	Body weight	kg	25.9	73	64
$AT_{non\text{-}carcinogenic}$	Averaging times	days	ED×365		
$AT_{carcinogenic}$			10×365	72.09×365	78.31×365

Table S5 The classification criteria of carcinogenic risk for health purpose [34].

CR	Level	Suitability of drinking water for health purpose
$<10^{-6}$	I	Very suitable for drinking water
1×10^{-6}-1×10^{-5}	II	suitable for drinking water
1×10^{-5}-1×10^{-4}	III	As drinking water should be paid attention to

1×10^{-4}-1×10^{-3}	IV	Safety measures should be taken as drinking water
$>1\times10^{-3}$	V	Not suitable for drinking water

Table S6. Descriptive statistics and drinking standard of potentially toxic elements in surface water (sample size = 34).

Parameters	Unit	Min	Max	Mean	Std	CV (%)	Standard	Exceeding
Fe	μg/L	120.00	1220.00	580.29	292.06	50.33	300	79.41%
Mn	μg/L	16.00	120.00	44.53	21.80	48.96	100	2.94%
Cu	μg/L	0.01	2.07	0.70	0.57	80.89	1000	0
Zn	μg/L	4.21	1660.00	111.16	305.37	274.72	1000	2.94%
Al	μg/L	74.00	560.00	288.35	111.70	38.74	200	79.41%
Hg	μg/L	0.03	0.81	0.39	0.18	46.42	1	0
As	μg/L	0.07	1.41	0.31	0.22	73.37	10	0
Se	μg/L	0.13	1.32	0.33	0.20	60.32	10	0
Cd	μg/L	0.01	4.13	0.87	1.39	159.42	5	0
Pb	μg/L	0.03	0.81	0.19	0.21	107.12	10	0
Li	μg/L	0.46	8.05	1.95	1.68	86.34	60	0
B	μg/L	0.14	16.10	4.39	4.56	103.83	500	0
Ba	μg/L	14.00	57.00	26.00	8.25	31.74	700	0
Sb	μg/L	0.04	0.75	0.18	0.17	94.78	5	0
Ni	μg/L	0.54	5.10	2.23	1.03	46.24	20	0
Co	μg/L	0.17	1.38	0.65	0.32	49.10	50	
Mo	μg/L	0.14	4.15	0.84	0.93	109.90	70	

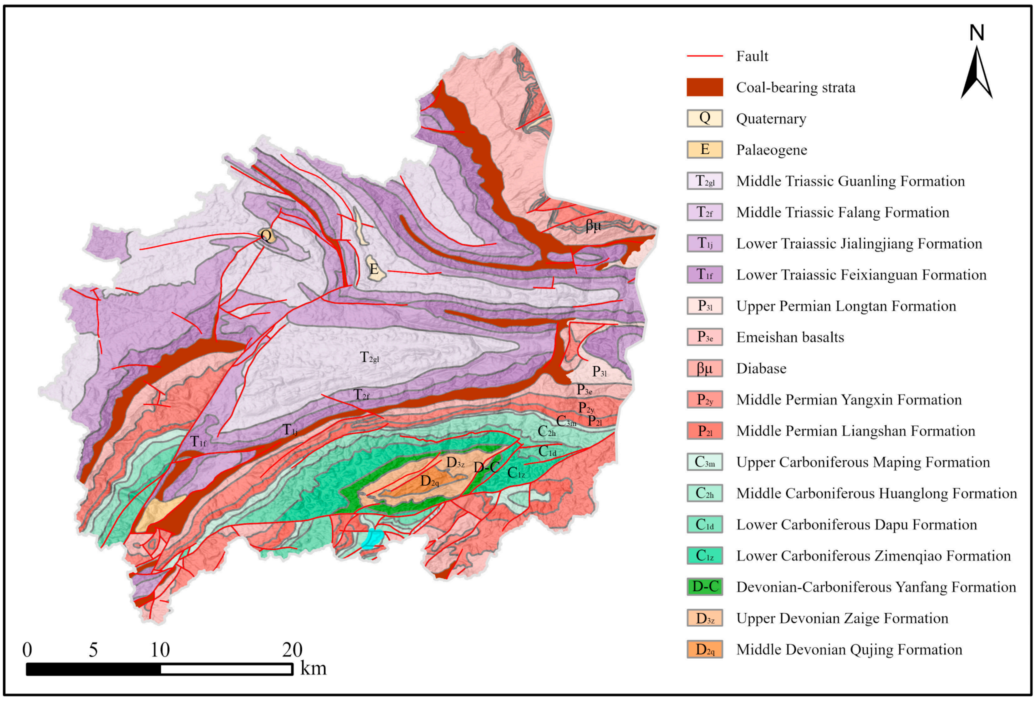

Figure S1. The geological map of the study area.

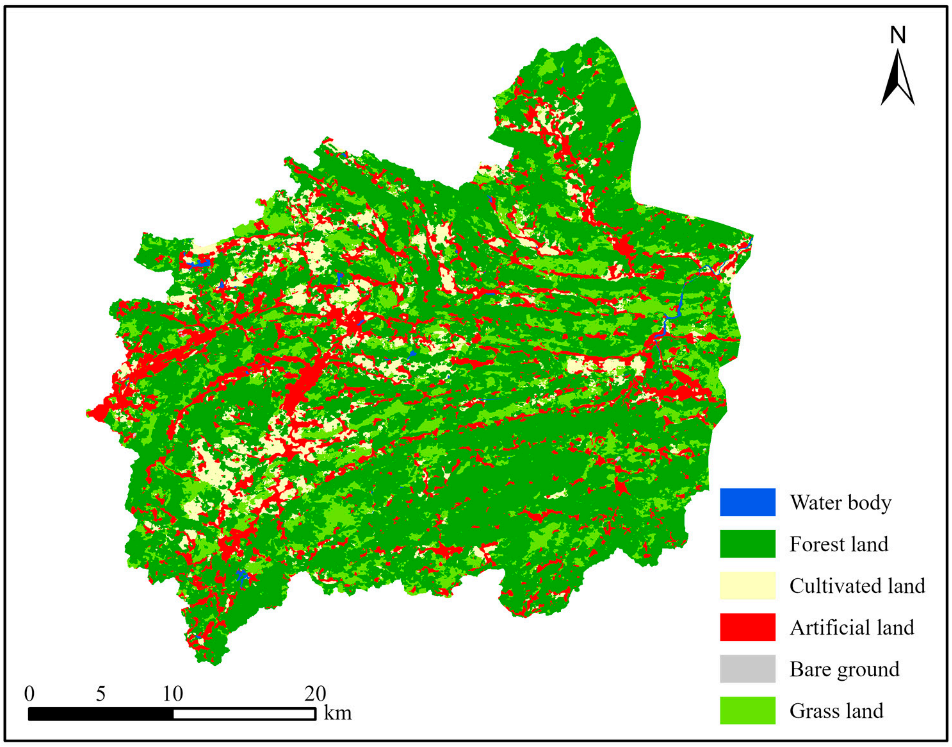

Figure S2. The land use map of the study area.

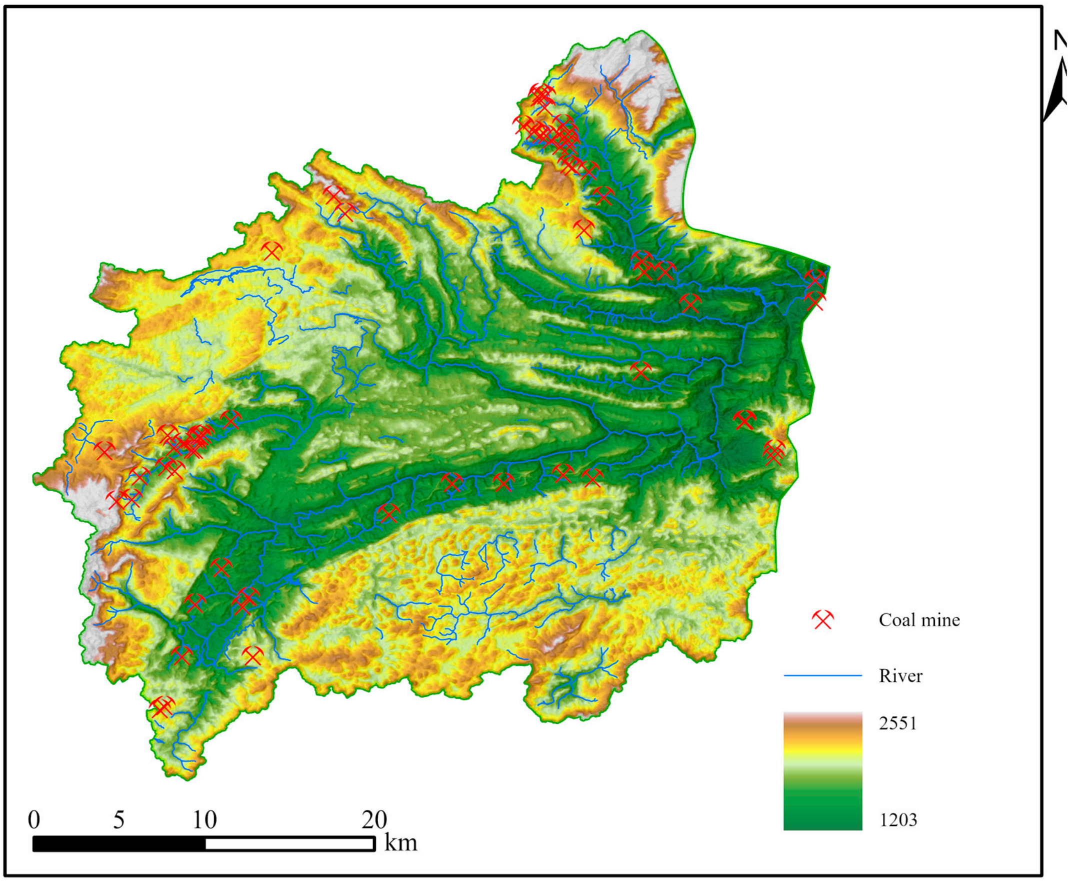

Figure S3. The location of coal mine in the study area.
